# IMAGINER 2—improving accuracy with augmented realIty navigation system during placement of external ventricular drains over Kaufman's, Keen's, Kocher's and Frazier's point

**DOI:** 10.3389/fsurg.2024.1513899

**Published:** 2025-01-21

**Authors:** Martin Vychopen, Fabian Kropla, Dirk Winkler, Erdem Güresir, Ronny Grunert, Johannes Wach

**Affiliations:** ^1^ Department of Neurosurgery, University Hospital Leipzig, Leipzig, Germany; ^2^ Medical Engineering, Frauenhofer Institute for Machine Tools and Forming Technology, Dresden, Germany

**Keywords:** external ventricular drain, augmented reality, Kaufman's point, Keen's point, Kocher's point, Frazier's points

## Abstract

**Background:**

External ventricular drain (EVD) placement is a routine neurosurgical procedure used to treat acute hydrocephalus and monitor intracranial pressure. Kocher's point is the most commonly used anatomical landmark, but other entry points can be challenging even for experienced neurosurgeons. Augmented reality (AR) may enhance the accuracy and safety of these procedures. Previous studies demonstrated improved ventriculostomy accuracy using AR among novices. This study evaluates AR's impact on EVD placement accuracy performed by experienced neurosurgeons.

**Methods:**

Eighteen neurosurgical experts performed ventriculostomies on a Styrofoam head model using Kaufman's, Keen's, Kocher's, and Frazier's points. Punctures were performed freehand (Freehand group) and with AR assistance (AR group). Post-procedure CT scans were used to compare the actual catheter tip positions with the ideal positions. Accuracy was assessed by the distance between real and ideal catheter tips and by Kakarla grading.

**Results:**

The AR group had a mean tip distance of 16.93 ± 9.38 mm compared to 21.71 ± 9.69 mm in the Freehand group (*p* = 0.003). The AR group also showed better Kakarla grading outcomes (Grade 1: *n* = 26, Grade 3: *n* = 26) vs. the Freehand group (Grade 1: *n* = 7, Grade 3: *n* = 53; *p* < 0.0001). Neurosurgeons with ≥7 years of experience demonstrated higher accuracy across both methods (*p* = 0.040).

**Conclusion:**

AR significantly enhances the accuracy of EVD placement, particularly using Kaufman's, Keen's, Kocher's, and Frazier's points, with experienced neurosurgeons benefiting the most from AR assistance.

## Graphical Abstract

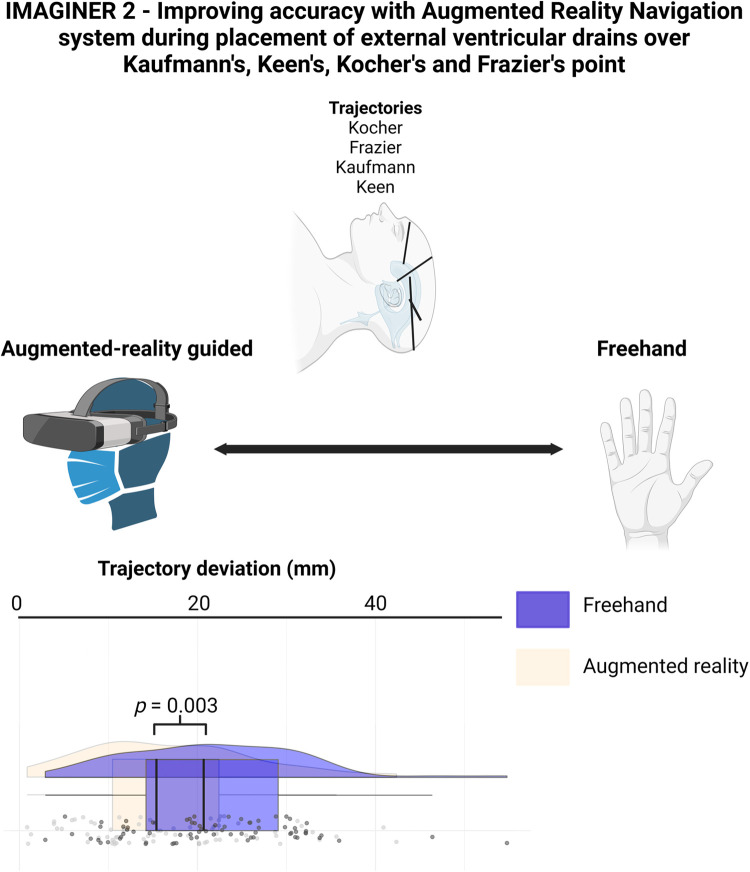

## 1 Introduction

Placement of external ventricular drainage (EVD) is a basic neurosurgical procedure performed on daily basis. Because of its crucial importance in patient care, the safety and accuracy is of paramount importance (1). In case of procedure-related complications such as misplacement or bleeding, the consequences might offset the benefits and even result in poor long-term prognosis (2). The contemporary standard of EVD placement is placement according to anatomical landmarks (3). In case of free-hand placement, increasing midline shift and left-sided placement of the catheter significantly worsen the accuracy according to Kakarla grade (4, 5). Kocher's point is the standard anatomical landmark used for the EVD placement. However, in case of slit ventricles or atypical ventricle configuration, accuracy decreases even in the hand of more experienced professionals (6). Occasionally (7), other entry points to the ventricles are used in order to safely perform the placement (8). We hypothesized that augmented reality (AR) might be a useful tool to help neurosurgeons identify the correct trajectory and navigate the puncture in order to reach the optimal catheter tip position. To perform the ventriculostomy with neuronavigation, acquisition costs can range from $350,000 up to $690,000. AR-guided placement might be a tool to provide much cheaper and effective solutions if high-end neuronavigation is not available (9). IMAGINER was an experimental study, which proved that neurosurgical-naïve medical students have higher accuracy in catheter placement if AR was used to aid the procedure (8). Based on the results of IMAGINER (10), we designed an experiment which compares the EVD placement performed according to anatomical landmarks (Freehand group) and with the AR (AR group).

## 2 Methods

### 2.1 Experimental design

#### 2.1.1 Preparation

We used Styrofoam heads for the experiment. This allowed us to avoid the use of cadavers or an animal model. The target points and optimal trajectories were virtually planned using Brainlab software (Brainlab AG, Munich). The planning was based on cranial magnetic resonance imaging (cMRI) scan of one of the authors (FK). After trajectory-planning was performed, an STL-file was obtained. This original file was then optimized in FREECad 1.0 software and uploaded to the Microsoft Hololens 2 (Microsoft Corporation, Redmond). The virtual model from this planning was then projected onto the Styrofoam heads. For the virtual model projection, the application was developed by our research team. Accurate surface registration was necessary to ensure precise overlay of the virtual model onto the Styrofoam heads. Using the Hololens 2, the manual surface-matching registration was performed with the Styrofoam head, similarly to our previous study IMAGINER (10).

#### 2.1.2 Puncture

One side of the Styrofoam head was punctured using Microsoft Hololens (AR group), while the other side was punctured without AR assistance (Freehand group). The participants were allowed to choose the sides freely. Entry points for the puncture were anteroposteriorly: Kaufmann's point, Kocher's point, Keen's point and Frazier's point (8). Kocher's point was anatomically defined as 12 cm superior and posterior from the nasion and 2.5–3 cm lateral to midline. Kaufmann's point placed 5 cm superior to the nasion and 3 cm lateral to midline. Keen's point 3 cm superior and posterior to the pinna of the ear, and Frazier's point 6 cm superior to the inion and 3 to 4 cm left or right to the midline (8).

Before the experiment, all neurosurgeons were given access to the MRI scan of the author to enhance their free-hand EVD placement. For AR-guided punctures, manual surface matching was performed using the Styrofoam head. The trajectories and entry points were pre-planned and projected onto the Styrofoam head. For anatomical punctures, neurosurgeons were allowed to reference the anatomical article describing the entry points and trajectories (8) prior to performing the puncture. Additionally, they were permitted to use a tape measure to identify the entry point.

We used steel needles to replace the EVD-catheters to perform the puncture of the Styrofoam model. The needles remained placed in the head for subsequent computed tomography (CT) scans. Before each puncture, the exact depth of needle insertion was determined due to missing haptic feedback. After the puncture was performed, we used plaster to permanently fix the needle in place and to make the face of the Styrofoam head recognizable in the CT scans.

#### 2.1.3 Evaluation

**Figure 1:**
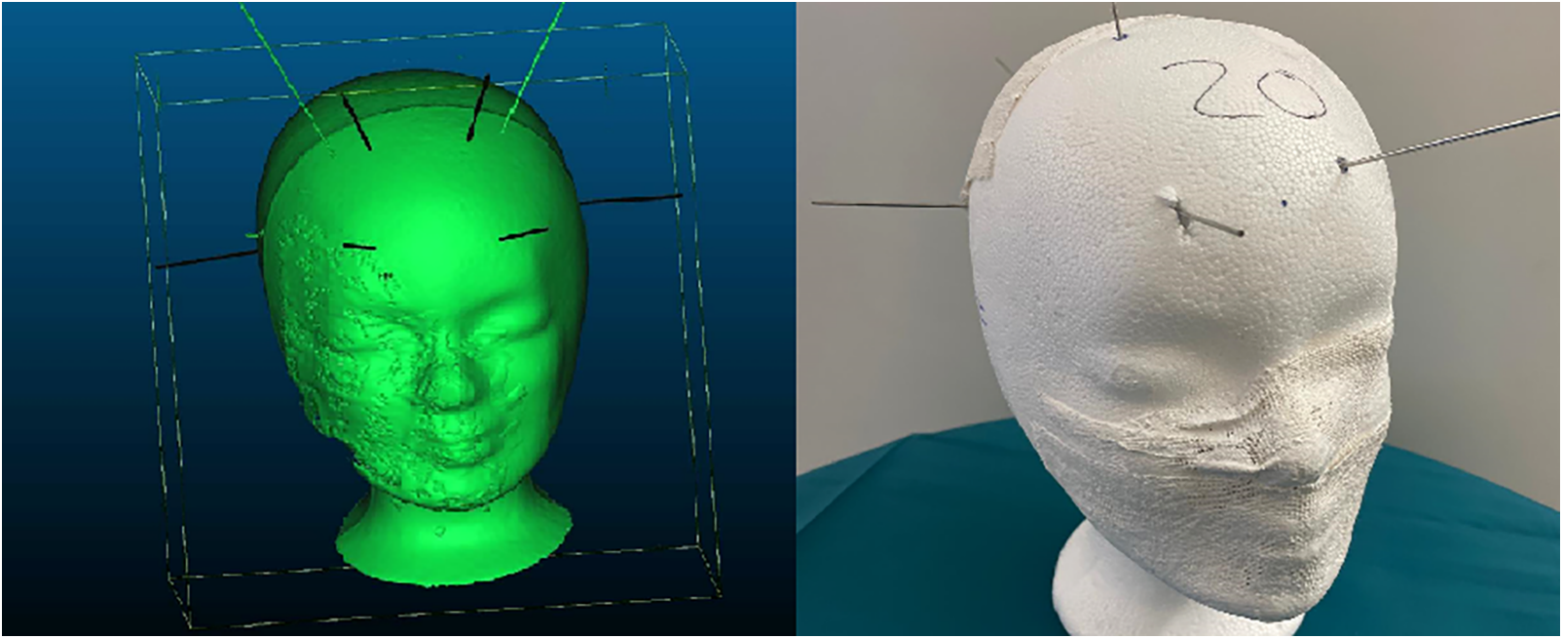
Punctured styrofoam head.

After performing the punctures, all model heads with inserted needles were scanned using CT (Figure 1). The resulting data were segmented using Brainlab software, identifying needles and skin based on grayscale values. The plaster represented the skin, and the needles indicated the actual trajectories.

##### 2.1.3.1 Distance comparison

An expected-actual comparison was conducted using ZEISS Inspect (Carl Zeiss GOM Metrology GmbH, Braunschweig). The virtual planning model (expected) was compared with the segmented Styrofoam model (actual). The evaluated data included the calculated trajectory deviation—distance between the expected and actual points measured in millimeters (mm).

##### 2.1.3.2 Accuracy according to Kakarla grading

**Figure 2:**
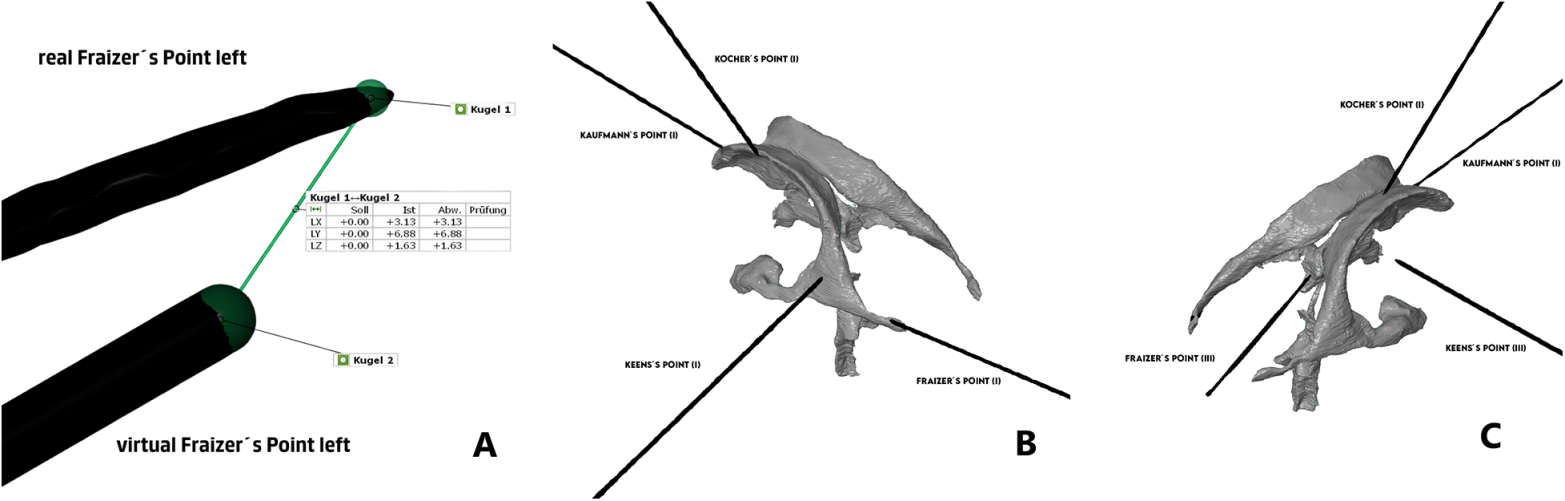
**(A)** Comparison of distance between catheter tips, **(B)** Kakarla evaluation of AR-group, **(C)** Kakarla evaluation of freehand group. This analysis focused on clinical aspect of the placement with main endpoint being the optimal intraventricular placement, ignoring the exact geometrical place of the target point.

Subsequently, we evaluated each trajectory (see Figure 2) separately according to Kakarla grading as 1-optimal placement, 2- suboptimal placement, in the wall of the ventricle with intraventricular tip, 3-extraventricular placement. For the sake of the analysis, we dichotomized the results as good (Kakarla 1) and suboptimal (Kakarla 2 and 3) (5).

##### 2.1.3.3 Accuracy according to experience

Finally, we divided neurosurgeons in experienced (>7 years of experience) and less experienced (≤7 years of experience).

### 2.2 Statistical analysis

Data were organized and analyzed using SPSS for Windows (version 29.0; IBM Corp., Armonk, NY, USA). We compared the means and standard-deviation of the geometrical placement using student *t*-test. All values with *p* < 0.05 were considered significant. For Data visualization, Raincloud-plot was constructed using R version 4.3.1 (R Foundation, Vienna, Austria) including the package ggrain.

## 3 Results

### 3.1 Experiment participant characteristics

18 neurosurgical experts took part in our experiment. Cumulatively, 144 punctures on 18 Styrofoam heads were performed. The participants had free choice of the side to use the AR, however, only one participant decided for the left-sided free-hand catheter placement.

### 3.2 Trajectory deviation

#### 3.2.1 Cumulative results

**Figure 3:**
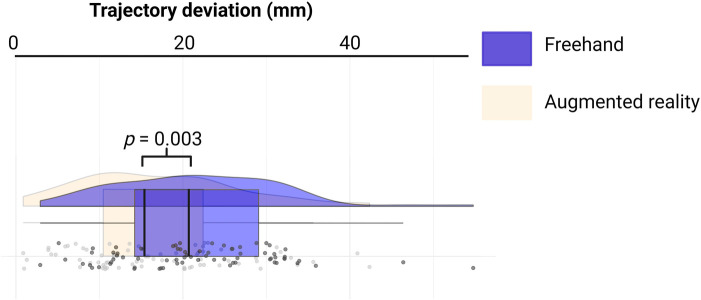
Rainbow-plot demonstrating the differences between the freehand and AR group based on trajectory deviation (mm).

The distance between the real and ideal catheter tip was 16.93 ± 9.38 mm in the AR group, compared to 21.71 ± 9.69 mm in the Freehand group, with a mean difference of 4.77 mm (see Figure 3, *p* = 0.003).

#### 3.2.2 Trajectory-based results

We subsequently compared all trajectories separately. The only statistically significant difference was observed in Keen's point trajectory (15.29 ± 7.27 mm vs. 23.48 ± 11.22 mm, *p* < 0.01). Punctions over Kocher's point showed no significant difference (16.33 ± 7.33 mm vs. 20.60 ± 6.88 mm, *p* < 0.08). All other trajectories showed no significant difference between AR group and Freehand group. For detailed information, see Table 1.

**Table 1:** Comparison of individual trajectories.

Anatomical point	AR group (mm) ± SD	Freehand group (mm) ± SD	*p* value
Fraizer	16.82 ± 10.28	20.03 ± 8.80	0.32
Kaufmann	19.29 ± 12.12	22.72 ± 11.20	0.38
Keen	15.29 ± 7.27	23.48 ± 11.22	0.01
Kocher	16.33 ± 7.33	20.60 ± 6.88	0.08

### 3.3 Kakarla grading

#### 3.3.1 Cumulative results—Kakarla grading

AR group showed significantly better results compared to Freehand group (*p* = 0.00001). After dividing the results in good (Kakarla = 1) and suboptimal (Kakarla = 2 and 3), AR group also shows superior results compared to Freehand group (Table 2, *p* = 0.0027).

**Table 2:** Evaluation according to Kakarla grading.

Kakarla grade	AR group (*n*)	Freehand group (*n*)
1	26	7
2	20	12
3	26	53

#### 3.3.2 Kakarla grading according to experience

We divided the trajectories in those performed by more experienced neurosurgeons (>7 years of experience) and less experienced neurosurgeons (≤7 years of experience). Both groups showed better results with AR (>7 years of experience, *p* < 0.0001, ≤7 years of experience *p* = 0.005).

Comparing all the trajectories cumulatively according to the experience, the experienced neurosurgeons performed better (*p* = 0.040). Table 3 summarizes the results.

**Table 3:** Kakarla grading data divided according to the years of experience.

Kakarla grade	AR group (≥7 YOE)	Freehand group (≥7 YOE)	AR group (<7 YOE)	Freehand group (<7 YOE)
1	17	4	9	3
2	10	9	10	3
3	9	23	17	30

## 4 Discussion

We performed an experiment evaluating Ventriculostomies on Styrofoam head performed by 18 neurosurgical experts using Kaufman's, Keen's, Kocher's and Frazier's point as entry point. We observed higher accuracy according to both trajectory deviation and Kakarla grading, if augmented reality was used.

### 4.1 Evaluation of the experts

We compared a ventriculostomy solely by neurosurgical experts. Because of the freedom to choose the side of the puncture, we have gathered very reliable data, as the majority preferred the right-sided placement of the needle. According to the published data, years of experience do impact the overall accuracy of the puncture. According to O'Neil et al. (11), senior residents and experienced neurosurgeons require less attempts in order to successfully place the catheter. Our data support the thesis that experienced surgeons not only perform better in overall evaluations regardless of the technique used but also benefit more significantly from AR support compared to their less experienced colleagues, particularly in achieving clinically optimal placement (Kakarla Grade 1). Specifically, experienced neurosurgeons achieved Kakarla Grade 1 placement in 21 cases (AR: *n* = 17; Freehand: *n* = 4), whereas less-experienced neurosurgeons achieved this in 12 cases (AR: *n* = 9; Freehand: *n* = 3).

As experience poses a significant limitation to the experiment, it is important to note that Kocher's point is the preferred entry point in our department. Additionally, two neurosurgeons with prior pediatric experience routinely use Frazier's point.

### 4.2 Design of experiment

The Styrofoam head models offered several advantages. They did not cause any CT-artefacts and have no restriction in availability compared to animal models or cadaveric heads. Furthermore, the material is easy to puncture and provides simultaneously sufficient support for the needle so that it does not accidentally dislocate.

Several disadvantages of the model presented themselves in the course of the experiment. The missing haptic feedback of ventricle puncture might have been a strong limitation (12). In case of successful ventricular puncture, a loss of resistance followed by CSF flow is a sign of success (13). Styrofoam head does not offer this feedback. If loss of resistance and CSF flow are missing in real ventriculostomies, it usually triggers a new puncture under alternated trajectory. This fact cannot be reflected in our experiment, as it only offers one single try without the possibility of correcting the needle trajectory.

### 4.3 Laterality

We observed a strong preference among neurosurgeons to right-sided anatomical placement of the catheter. This reflects the daily clinical practice, as the right-sided puncture is often the preferred approach (14). This preference should theoretically strengthen the results and supports higher accuracy in the Freehand group. However, we noted a very high number of Kakarla Grade 3 catheter tips. This suggests, that despite of the complexity and obvious disadvantage of left-sided puncture, AR guided catheter placement may be superior to Freehand group, even in hands of experienced professionals (15, 16).

### 4.4 Evaluation

We performed two types of evaluation; trajectory deviation and target point evaluation according to Kakarla grading. Both have some pros and cons.

Trajectory deviation offers a comparison with ideal trajectory as planned preoperatively. This offers an exact comparison of catheter tip distance to preoperatively planned target point. However, it misses the clinical critically important information—the position of the catheter according to the ventricular system.

Evaluation according to Kakarla scale (5) presents clinically valuable evaluation of actual catheter tip placement in relation to the ventricle. On the other hand, it omits the geometry and does not evaluate the deviation from the ideal trajectory.

### 4.5 Augmented-reality

Several possibilities to navigate the EVD placement have already been analyzed (17). The complexity of use and mainly retrospective, single center reports with strongly biased data limit the routine use of this navigation techniques. Under such circumstances, a simple AR-based tools might offer the most straightforward solution.

### 4.6 Limitations

The experiment-design had several limitations. The absence of haptic feedback and CSF flow, which in real-life puncture would prompt the neurosurgeon to correct the trajectory and perform a new puncture, likely contributed to higher rates of Kakarla Grade 3 (intraparenchymal) placements. In real-life scenarios, the missing CSF flow and loss of resistance usually indicate a suboptimal catheter tip position, triggering a new attempt with a corrected trajectory. The second factor contributing to high grade of Kakarla 3 rate is the fact that a model MRI used for the experiment design was performed on young individual without any sign of hydrocephalus and radiologically relatively slit ventricle-system. For the detailed image of the MRI, please see the Supplementary File.

## 5 Conclusion

Our study experimentally demonstrates higher accuracy in EVD placement using Kaufman's, Keen's, Kocher's and Frazier's points when AR was used. However, despite good results, we still see a high number of missed attempts in both groups. In the future, further development and simplification of the tools might play a crucial role in order to minimize missed target points.

## Data Availability

The original contributions presented in the study are included in the article/Supplementary Material, further inquiries can be directed to the corresponding author.
