# Supplementary figures and images for: IMAGINER 2—improving accuracy with augmented realIty navigation system during placement of external ventricular drains over Kaufman's, Keen's, Kocher's and Frazier's point
Source: Front Surg. 2025 Jan 21;11:1513899. doi: 10.3389/fsurg.2024.1513899 (PMC11790646; doi:10.3389/fsurg.2024.1513899)

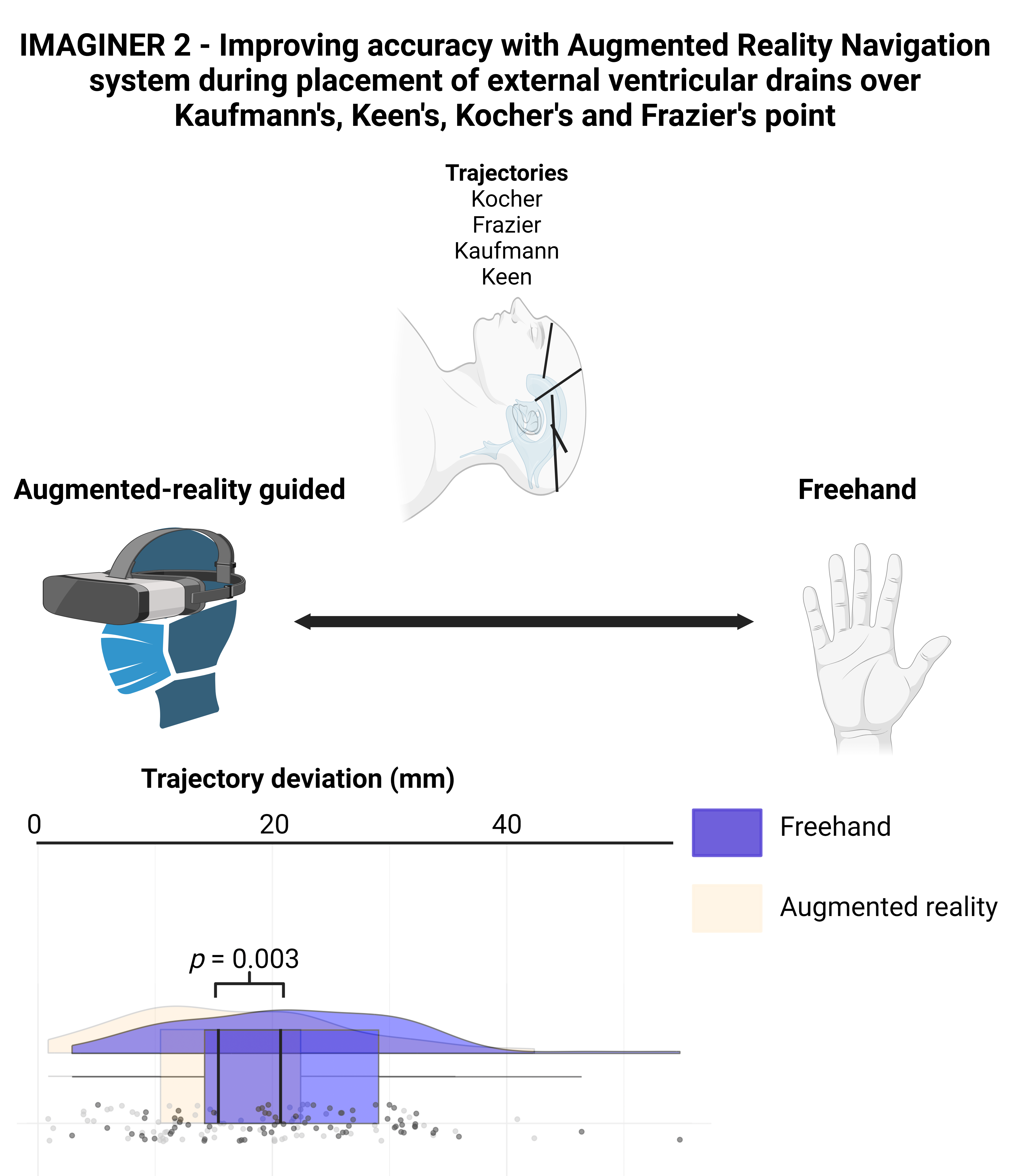

Supplement: Supplementary file 1 [file Image1.tif]

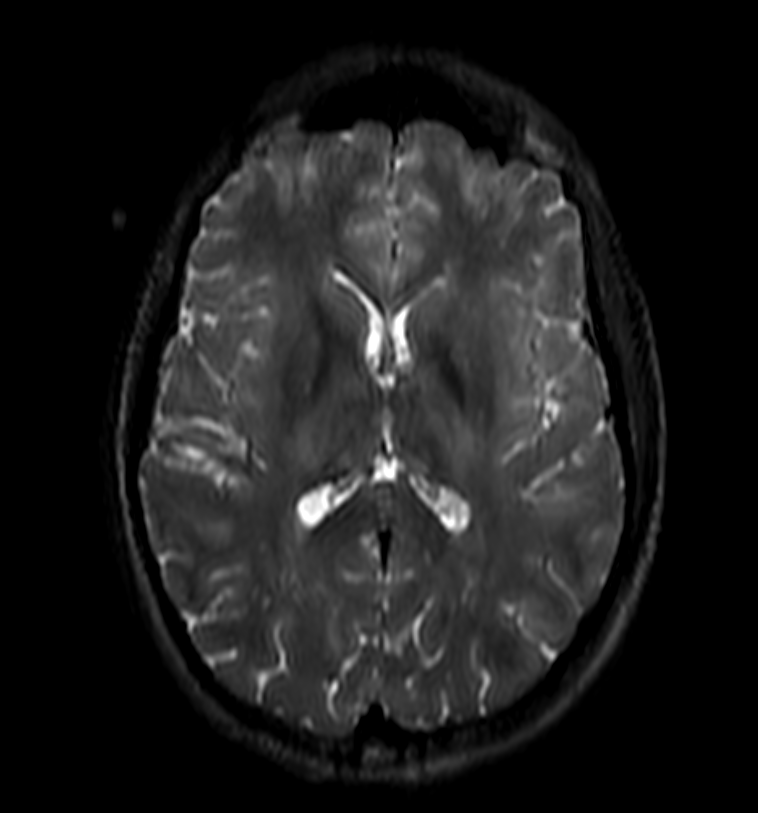

Supplement: Supplementary file 2 [file Image2.png]
